# The evaluation of the outcome in myelodysplastic patients by using non-cytogenetic prognostic scores

**Published:** 2014-09-25

**Authors:** O Căzăceanu, AM Vlădăreanu, H Bumbea, M Begu, M Onisai, C Enache

**Affiliations:** University Emergency Hospital, Department of Hematology, Bucharest, Romania

**Keywords:** myelodysplastic syndrome, prognostic score, acute myeloid leukemia

## Abstract

Abstract

Myelodysplastic syndromes (MDS) are a heterogeneous group of clonal hematopoietic stem cell disorders; they are characterized by ineffective hematopoiesis and a predilection to the development of acute myeloid leukemia (AML). For a rapid evaluation of the outcome in myelodysplastic patients non-cytogenetic prognostic scores can be used.

Aim: This study proposed to demonstrate that age and gender are important factors in the outcome of the patients diagnosed with myelodysplastic syndrome.

Materials and methods: This study was conducted in the Department of Hematology of the Emergency University Hospital Bucharest during October 2008 and October 2012.

Results: Male sex and age higher than 60 years are associated with high risk in the studied cases by using the Spanish prognostic score. According to Goasguen score: male sex and age, patients older than 60 years, present characteristics associated with an intermediate risk. Based on the Dusseldorf score, age over 60 years and female gender were associated with pronounced risk in the examined group. By examining the Bournemouth score in our group, we found that age > 60 years correlated with a higher frequency of risk, but no significant differences regarding the sex of patients were observed.

Conclusions: We concluded that age > 60 years and male gender are important predisposing factors in the survival.

Abbreviations MDS = myelodysplastic syndromes, AML = acute myeloid leukemia, LDH = lactate dehydrogenase, FAB = French-American-British, WHO = World Health Organization, IPSS = International Prognostic Scoring System, ALIP = abnormal localization of immature precursors, WPSS = WHO classification-based prognostic scoring system, FISH = fluorescence in situ hybridization, del = deletion.

## Introduction

Myelodysplastic syndromes (MDS) are a heterogeneous group of clonal hematopoietic stem cell disorders and they are characterized by ineffective hematopoiesis, morphologic abnormalities in one or more cell lines in an usually cellular bone marrow and a predilection to the development of acute myeloid leukemia (AML) [**1,2**]. A succession of MDS classification systems has been developed to facilitate the prediction of the risk of progression to AML and overall survival [**2-5**].

 For a rapid evaluation of the outcome in myelodysplastic patients, non-cytogenetic prognostic scores can be used. The following prognostic scores are not based on a cytogenetic analysis and they are reliable to evaluate recently diagnosed patients with myelodysplastic syndrome: Spanish score [**6**], Goasguen score [**7**], Dusseldorf score [**8**] and Bournemouth score [**9,10**].

 Aim: Our aim was to demonstrate that we can still relay on non-cytogenetic prognostic scores to evaluate patients who were diagnosed with myelodysplastic syndrome, and that age and gender are important factors in the outcome of these patients.

 Matherials and method: This study was conducted in the Department of Hematology of the Emergency University Hospital Bucharest during October 2008 and October 2012. 110 patients were enrolled; only patients at diagnose were evaluated. The following tests were performed for each patient: cells blood count, bone marrow smear (to evaluate the percent of myeloblasts) and level of lactate dehydrogenase (LDH).

 Results: The Spanish score is calculated based on the following parameters: percent of bone marrow blasts, platelets number and patient age. Male sex and age higher than 60 years are associated with high-risk in the studied cases (**Table 1,2**).

**Table 1:** Distribution of patients based on Spanish score and age

		Patient age		Total
		Younger than 60 years	Older than 60 years	
	Low risk	15	42	57
Spanish score	Intermediate risk	5	35	40
	High risk	1	12	13
Total		21	89	110

**Table 2:** Distribution of patients based on Spanish score and gender

		Patient sex		Total
		female	male	
	Low risk	32	25	57
Spanish score	Intermediate risk	19	21	40
	High risk	4	9	13
Total		55	55	110

 Another prognostic score which does not use the cytogenetic analysis is Goasguen score and it is based on: hemoglobin level, platelets number and percent of bone marrow blasts. According to this score: male sex and age, patients older than 60 years, present characteristics associated with an intermediate risk (**Table 3,4**).

**Table 3:** Distribution of patients based on Goasguen score and gender

		Patient sex		Total
		female	male	
	Low risk	30	23	53
Goasguen score	Intermediate risk	25	32	57
Total		55	55	110

**Table 4:** Distribution of patients based on Goasguen score and age

		Patient age		Total
		Younger than 60 years	Older than 60 years	
	Low risk	12	41	53
Goasguen score	Intermediate risk	9	48	57
Total		21	89	110

 Dusseldorf prognostic score is calculated based on the percent of marrow blasts, LDH, hemoglobin level and platelets number. 43.5% of the evaluated patients were classified with intermediate risk and 34.5% with high risk. Age over 60 years and female gender were associated with pronounced risk in the examined group (**Table 5,6**)..

**Table 5:** Distribution of patients based on Dusseldorf score and age

		Patient age		Total
		Younger than 60 years	Older than 60 years	
	Low risk	4	7	11
Dusseldorf score	Intermediate risk	11	50	61
	High risk	6	32	38
Total		21	89	110

**Table 6:** Distribution of patients based on Dusseldorf score and gender

		Patient sex		Total
		female	male	
	Low risk	5	6	11
Dusseldorf score	Intermediate risk	33	28	61
	High risk	17	21	38
Total		55	55	110

**Fig. 1:**
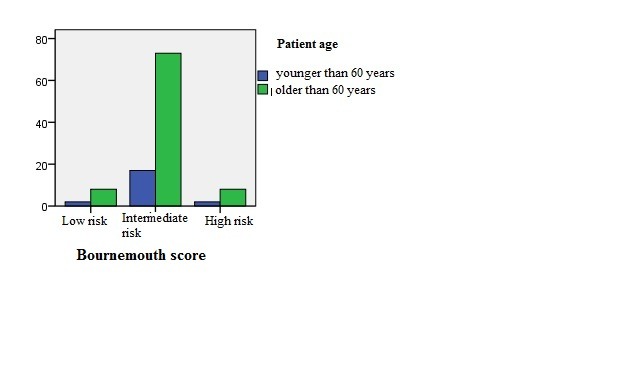
Graphic representation of Bournemouth score and age

**Fig. 2:**
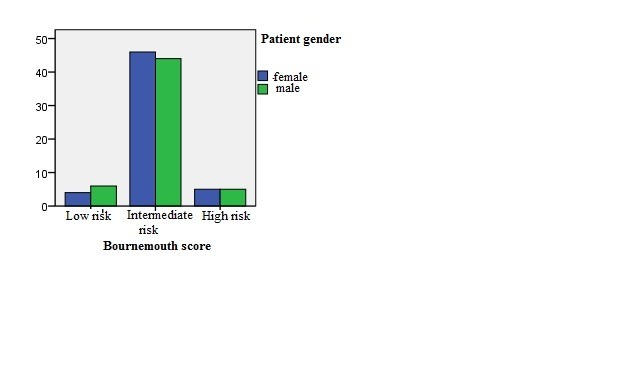
Graphic representation of Bournemouth score and gender

 Bournemouth score takes into account the percent of bone marrow blasts, neutrophils, platelets number and hemoglobin level. By examining this score we found that age > 60 years correlated with a higher frequency of risk, but no significant differences regarding the sex of patients were observed in our group (**Fig. 1,2**).

 From the 110 patients enrolled in this study, only 25 evolved to acute myeloid leukemia. This group consisted of 9 women and 16 men; at the time of diagnosis of acute leukemia, five of them were aged under 60 years and twenty over 60 years (**Table 7**).

**Table 7:** Distribution of patients who evolved from myelodysplastic syndrome to acute myeloid leukemia

Patients age	Number of patients	Patients sex	Number of patients	Percent
Younger than 60 years	5	Female	9	36,0
Older than 60 years	20	Male	16	64,0
Total	25	Total	25	100,0

 40% of the female and 75% of the male patients who evolved to acute 7 leukemia have the most severe prognostic risk demonstrated by applying all 4 scores above.

 In the group of patients older than 60 years: 80.76% of them transformed from a myelodysplastic entity into AML, and 23/25 died. In the other group, with patients younger than 60 years, 25% of the patients evolved to AML and 3/5 died by the end of the study.

## Discussions

 Over the past 25 years, diagnostic criteria have been set up to diagnose the MDS: 2 classification systems (French-American-British [FAB] and World Health Organization [WHO]) and several prognostic-scoring systems, the most common being the International Prognostic Scoring System (IPSS), have been widely used [**11**].

 Regarding the clinical outcomes, other studies have suggested the importance of a variety of clinical features, including different numbers or types of cytopenias (less than 1 or 2 cytopenias, thrombocytopenia, anemia versus neutropenia) [**12**], bone marrow blast percentages [**13,15**], and cytogenetic abnormalities [**4,16**]. Because the vast majority of primary MDS patients are elderly, age-stratified morbidity and mortality figures are needed regarding their clinical outcome [**4**]. Our data are similar to those previously reported, showing that age > 60 years is an important factor associated with poor prognosis and survival.

 Both myelodysplasia and myeloproliferative diseases are uncommon in childhood, perhaps because small series of patients are the norm [**1**]. When all the patients were included in the analysis, sex, age, the proportion of blasts in blood or bone marrow, or the presence of abnormal localization of immature precursors (ALIP) in the bone marrow trephine were not of prognostic significance [**1**]. Our study revealed that male sex is more frequently associated with severe prognostic risk compared to female gender.

 Cytogenetic findings have an established role in the diagnosis and assessment of prognosis of MDS and are emerging as an important factor in the treatment selection and monitoring response to therapy [**2**]. Unfortunately, the IPSS system underweights the clinical importance of severe (life-threatening) neutropenia and thrombocytopenia, in determining the need for therapeutic intervention. Although imperfect in its clinical utility, the IPSS has been very useful in examining and comparing the outcomes of clinical trials [**11**]. Since its publication, its utility has been confirmed in many institutions and refinements of the IPSS (e.g. the WHO classification-based prognostic scoring system, WPSS) continue to be proposed [**11,17**]. 

In addition, cytogenetic subgroups and prognostic variables have recently been suggested as providing improved prognostic evaluation of clinical outcomes of primary MDS patients [**18,20**]. Although the established prognostic scoring systems are based on conventional cytogenetics, some studies showed that chromosomal abnormalities detected by fluorescence in situ hybridization (FISH) may provide prognostic information [**21,22**], and may be useful in supporting the clinical decision making in the selected cases, such as those with del(5q) or with del(7q) or monosomy 7 [**23**].

## Conclusions

 The diagnosis of myelodysplastic syndromes is a multi-step procedure and the evaluation of the outcome of these patients can sometimes be difficult to assess. Using simple laboratory tests, such as cells blood count, bone marrow smears and level of lactate dehydrogenase, 4 non-cytogenetic prognostic scores can be applied to obtain important information about the risk assessment of the patients. By the end of this study, we concluded that age > 60 years and male gender are important predisposing factors in survival.
